# Computing SARS-CoV-2 Infection Risk From Symptoms, Imaging, and Test Data: Diagnostic Model Development

**DOI:** 10.2196/24478

**Published:** 2020-12-16

**Authors:** Christopher D'Ambrosia, Henrik Christensen, Eliah Aronoff-Spencer

**Affiliations:** 1 Department of Computer Science and Engineering University of California San Diego San Diego, CA United States; 2 Division of Infectious Diseases and Global Public Health School of Medicine University of California San Diego San Diego, CA United States

**Keywords:** health, informatics, computation, COVID-19, infection, risk, symptom, imaging, diagnostic, probability, machine learning, Bayesian, model

## Abstract

**Background:**

Assigning meaningful probabilities of SARS-CoV-2 infection risk presents a diagnostic challenge across the continuum of care.

**Objective:**

The aim of this study was to develop and clinically validate an adaptable, personalized diagnostic model to assist clinicians in ruling in and ruling out COVID-19 in potential patients. We compared the diagnostic performance of probabilistic, graphical, and machine learning models against a previously published benchmark model.

**Methods:**

We integrated patient symptoms and test data using machine learning and Bayesian inference to quantify individual patient risk of SARS-CoV-2 infection. We trained models with 100,000 simulated patient profiles based on 13 symptoms and estimated local prevalence, imaging, and molecular diagnostic performance from published reports. We tested these models with consecutive patients who presented with a COVID-19–compatible illness at the University of California San Diego Medical Center over the course of 14 days starting in March 2020.

**Results:**

We included 55 consecutive patients with fever (n=43, 78%) or cough (n=42, 77%) presenting for ambulatory (n=11, 20%) or hospital care (n=44, 80%). In total, 51% (n=28) were female and 49% (n=27) were aged <60 years. Common comorbidities included diabetes (n=12, 22%), hypertension (n=15, 27%), cancer (n=9, 16%), and cardiovascular disease (n=7, 13%). Of these, 69% (n=38) were confirmed via reverse transcription-polymerase chain reaction (RT-PCR) to be positive for SARS-CoV-2 infection, and 20% (n=11) had repeated negative nucleic acid testing and an alternate diagnosis. Bayesian inference network, distance metric learning, and ensemble models discriminated between patients with SARS-CoV-2 infection and alternate diagnoses with sensitivities of 81.6%-84.2%, specificities of 58.8%-70.6%, and accuracies of 61.4%-71.8%. After integrating imaging and laboratory test statistics with the predictions of the Bayesian inference network, changes in diagnostic uncertainty at each step in the simulated clinical evaluation process were highly sensitive to location, symptom, and diagnostic test choices.

**Conclusions:**

Decision support models that incorporate symptoms and available test results can help providers diagnose SARS-CoV-2 infection in real-world settings.

## Introduction

Despite advances in molecular diagnostics and imaging, ruling in or ruling out COVID-19 infection in an individual patient remains a significant challenge [1]. Current guidelines from the Centers for Disease Control and Prevention (CDC) recommend providers to determine whether signs or symptoms are compatible with COVID-19 infection and to test appropriate patients using nucleic acid amplification tests (NAATs) or antigen detection assays [2]. However, the diverse clinical presentations of COVID-19 infection may mimic those of common infections, potentially confounding the diagnostic value of presenting symptoms [3]. Moreover, significant and evolving differences in estimated local disease prevalence for both COVID-19 infection and seasonal respiratory illnesses meaningfully impact differential diagnostic probabilities. Despite the uncertain utility of this symptom and local prevalence information, in low-resource and community settings such as ambulatory clinics, nursing homes, and telemedicine, these may be the only sources of data. In higher-resource settings, NAATs [4], antibody based lateral flow assays [5], chest radiography (CXR) [6], and computed tomography (CT) [7] may be available, yet published literature notes varied performance. Despite these limitations, clinicians with access to any imaging and testing modalities must optimize diagnostic imaging and testing sequences to appropriately reduce diagnostic uncertainty in a given setting.

This complexity highlights the need for reliable and user-friendly clinical decision support systems (CDSS) that suggest optimal testing strategies and quantify SARS-CoV-2 infection risk for patients across the continuum of care. Prior research has demonstrated the potential utility of Bayesian inference [8,9] and machine learning [10,11] methods in diagnostic decision making, but computational clinical decision support has often been underutilized due to a lack of accessibility, transparency, workflow integration, and most importantly, the flexibility to incorporate local provider beliefs into the diagnostic model [12,13].

A robust diagnostic risk model should be built on individualized patient data that is easily obtained by patients and health care workers. Menni et al [14] analyzed a large database of smartphone-enabled, self-reported symptom tracker records to predict potential COVID-19 cases using logistic regression models. In the US test set, this approach had a reported sensitivity of 66% and a specificity of 83%. Ahsan et al [15] used deep learning techniques to differentiate between COVID-19 and non–COVID-19 patients based on open-source imaging and clinical data. However, the performance of this or other machine learning models in clinical settings has not yet been examined.

Moreover, in evolving contexts where illness presentation may change depending on host and viral characteristics, large databases of individual patient records may not be available or locally relevant. Constructing inflexible predictive algorithms, such as logistic regression models, based on out-of-date and locally irrelevant data sets would significantly compromise diagnostic accuracy. Addressing these issues, Chishti et al [16] demonstrated the advantages of using flexible probabilistic frameworks built without large-scale clinical data sets to generate ranked differential diagnoses that are more accurate that those developed by physicians.

Combining the approaches of this prior work suggests that an appropriate diagnostic support model should rely on easily obtained symptom data, probabilistic frameworks to avoid the need for large-scale data sets, and most importantly, a flexible schema to refine predictions based on provider judgment and the ability to adapt to changes in local prevalence and current diagnostic test performance. To this end, we present a comparison and clinical validation of 3 such quantitative models as well as an ensemble approach to the diagnosis of COVID-19 in ambulatory and acute care settings. We then illustrate how this approach can be employed to help providers optimally reduce diagnostic uncertainty through appropriate diagnostic test choices and update predictions based on local clinical context and test results as that are obtained. Finally, we provide an interactive, online resource to assess COVID-19 infection probability based on user-defined parameters such as local disease prevalence, imaging, and testing performance [17].

## Methods

### Data Acquisition

National and state-specific confirmed cases of COVID-19 as of July 2, 2020, were acquired from the Center for Systems Science and Engineering at Johns Hopkins University [18]. During our model training, validation, and testing process, we assumed a national SARS-CoV-2 infection prevalence of 11.1% based on the total confirmed count of 5,438,325 in the United States as of August 17, 2020 [18], a population estimate of 328,239,523 [19], and an estimated reporting rate of 14.9% [20-22]. Prevalence and conditional symptom probabilities for diseases in the differential diagnosis were collected from the CDC and literature estimates (Multimedia Appendix 1, Table S1). COVID-19 symptom probabilities were developed primarily from a 393-person consecutive patient series [23] and supplemented by 3 meta-analyses, which included 3062 [24], 49,504 [25], and 53,000 patients [26]. Where conditional symptom probabilities have not been described in the literature, we used a symptom probability of 1.0% based on our assumption that a higher conditional symptom probability would have been discussed in the literature.

To incorporate location and diagnostic test results into risk predictions, we used state-level case figures [18], state-level population data [19], and the estimated reporting rate [20-22] to compute an estimated SARS-CoV-2 infection prevalence for each state. We sourced imaging diagnostic accuracies from existing literature [6,7] and laboratory test accuracies from the Johns Hopkins Center for Health Security. The reverse transcription-polymerase chain reaction (RT-PCR) sensitivity of 70% is based on published estimates [4] that take into account operator dependency and variability in viral load across upper respiratory tract sites [27]. The RT-PCR specificity of 99.8% is based on published data from Abbott Molecular [28]. Antibody test sensitivity and specificity are based on published figures [5] for electro-chemiluminescence immunoassay completed between 0-6 days of infection. We computed likelihood ratios and prevalence-adjusted predictive values based on sensitivity, specificity, and our estimated national COVID-19 prevalence of 11.1% (Table 1).

**Table 1:** Imaging and laboratory diagnostic test statistics for SARS-CoV-2 infection.

Diagnostic test	Sensitivity (%)	Specificity (%)	Likelihood ratio (%)		Predictive value^a^ (%)	
			Positive	Negative	PPV^b^	NPV^c^
Chest radiography	69.0 [6]	50.0^d^	1.4	0.6	14.7	92.8
Computed tomography [7]	97.0	57.3	2.3	0.1	22.1	99.3
RT-PCR^e^	70.0 [4]	97.0 [28]	23.3	0.3	74.5	96.3
Antibody (0-6 days) [5]	65.5	99.8	327.5	0.3	97.6	95.9

^a^Prevalence-adjusted predictive values assume a prevalence of 11.1%.

^b^PPV: positive predictive value.

^c^NPV: negative predictive value.

^d^No published figures available.

^e^RT-PCR: reverse transcription-polymerase chain reaction.

### Training

We developed Bayesian inference network (BN) and set-cover (SC) models from published disease prevalence and conditional symptom probabilities (see Multimedia Appendix 1, Table S1). We simulated symptom profiles and diagnoses for 100,000 patients using the published aggregate diagnosis prevalence and conditional symptom probabilities. Of the 100,000 simulated patients, the number of patients assigned to each mutually exclusive diagnosis was proportional to diagnosis prevalence. Within each diagnostic class, we simulated a joint symptom distribution by randomly assigning the presence or absence of each symptom to every patient. The number of patients with a positive symptom within each diagnostic class was proportional to the conditional symptom probability for that symptom and diagnosis. We trained our distance metric learning (DML) and ensemble models on this simulated data.

### Study Design

We analyzed consecutive ambulatory and hospitalized patients with COVID-19–compatible syndromes presenting to University of California San Diego Medical Center over 14 days in March and April 2020, with institutional review board approval (#200498). Patients were included if they had a recorded presenting illness including fever or cough, and at least a single NAAT in the electronic health record. Patients were labeled “positive” if they had one or more positive RT-PCR tests and a compatible syndrome or findings on radiographic imaging. Patients were labeled “negative” if they had 2 or more consecutive negative NAAT tests (>72 hours apart) or a single negative RT-PCR and a negative antibody test within 14-21 days of symptom onset. Chart review was performed manually by an infectious disease specialist with an anonymized and blinded data set presented for analysis (see Multimedia Appendix 1 for additional details).

### Data Analysis

We calculated the sensitivity, specificity, and prevalence-adjusted accuracy as well as the prevalence-adjusted negative predictive value (NPV) and positive predictive value (PPV) of each model on the clinical test data using standard Wald-type CIs [29]. We estimated the 95% CIs for sensitivity and specificity using Clopper-Pearson exact binomial proportion CIs [29]. We estimated 95% CIs for accuracy using the normal approximation method [29]. For the imaging and laboratory tests, we computed likelihood ratios based on sensitivity and specificity; and prevalence-adjusted predictive values based on sensitivity, specificity, and an assumed national COVID-19 prevalence of 11.1%.

## Results

### Patient Characteristics

In total, 55 individuals and the presence or absence of 13 symptoms at initial presentation were included in our clinical test data set. Of this, 38 patients (69.1%) were confirmed SARS-CoV-2 infection positive by RT-PCR; 44 subjects were seen via inpatient services, and 11 were seen as outpatients. The majority of subjects (n=43, 78.2%) presented with fever, 63.6% (n=35) with shortness of breath or dyspnea, 54.5% (n=30) with nonproductive cough, 21.8% (n=12) with productive cough, 50.9% (n=28) with fatigue or exhaustion, 9.1% (n=5) with loss of smell, 7.3% (n=4) with sore throat or pharyngalgia, 18.2% (n=10) with body or muscle aches, 16.4% (n=9) with headaches, 16.4% (n=9) with diarrhea, 14.5% (n=8) with nausea, 5.5% (n=3) with vomiting, and 3.6% (n=2) with nasal congestion or rhinorrhea (Table 2; Multimedia Appendix 1, Table S2).

**Table 2:** Clinical test data set: patient characteristics.

Characteristic		Total, n (%)	SARS-CoV-2 test result	
			Positive, n (%)	Negative, n (%)
Patients		55 (100)	38 (69)	17 (31)
**Sex**				
	Male	27 (49)	21 (55)	6 (35)
	Female	28 (51)	17 (45)	11 (65)
**Age (years)**				
	<60	27 (49)	19 (50)	8 (47)
	60-70	13 (24)	9 (24)	4 (24)
	70-80	9 (16)	5 (13)	4 (24)
	>80	6 (11)	5 (13)	1 (6)
**Setting**				
	Inpatient	44 (80)	32 (84)	12 (71)
	Outpatient	11 (20)	6 (16)	5 (29)
**Symptoms**				
	Fever	43 (78)	33 (87)	10 (59)
	Dyspnea	35 (64)	33 (87)	2 (12)
	Dry cough	30 (55)	28 (74)	2 (12)
	Productive cough	12 (22)	4 (11)	8 (47)
	Fatigue	28 (51)	21 (55)	7 (41)
	Loss of smell	5 (9)	5 (13)	0 (0)
	Sore throat	4 (7)	1 (3)	3 (18)
	Body/muscle aches	10 (18)	8 (21)	2 (12)
	Headache	9 (16)	6 (16)	3 (18)
	Diarrhea	9 (16)	5 (13)	4 (24)
	Nausea	8 (15)	3 (8)	5 (29)
	Vomiting	3 (6)	1 (3)	2 (12)
	Nasal congestion/rhinorrhea	2 (4)	0 (0)	2 (12)
**Comorbidities**				
	Cancer	9 (16)	5 (13)	4 (24)
	Diabetes	12 (22)	10 (26)	2 (12)
	Cardiovascular disease	7 (13)	4 (11)	3 (18)
	Hypertension	15 (27)	12 (32)	3 (18)

### Classification Performance in the Clinical Test Data Set

Base models classified SARS-CoV-2 infection in the clinical test data set with sensitivities and specificities of 81.6% (95% CI 65.7 to 92.3) and 58.8% (95% CI 32.9 to 81.6) for the BN model; 0.0% (95% CI 0.0 to 9.3) and 100.0% (95% CI 80.5 to 100.0) for the SC model; 84.2% (95% CI 68.7 to 94.0) and 64.7% (95% CI 38.3 to 85.8) for the DML model; and 81.6% (95% CI 65.7 to 92.3) and 70.6% (95% CI 44.0 to 89.7) for the ensemble model. The overall accuracy of each of these models was 61.4% (95% CI 48.5 to 74.2) for the BN model; 88.9% (95% CI 80.6 to 97.2) for the SC model; 66.9% (95% CI 54.4 to 79.3) for the DML model; and 71.8% (95% CI 59.9 to 83.7) for the ensemble model. The prevalence-adjusted positive and negative predictive values for each model were 19.9% (95% CI 10.5 to 29.2) and 96.2% (95% CI 92.5 to 100.0) for the BN model; 0.0% and 88.9% for the SC model; 23.0% (95% CI 11.3 to 34.7) and 97.0% (95% CI 93.8 to 100.0) for the DML model; and 25.8% (95% CI 11.4 to 40.2) and 96.8% (95% CI 93.7 to 100.0) for the ensemble model (Table 3).

**Table 3:** Classification performance on the clinical test data set for the developed base and ensemble models compared to a logistic regression model reported in the literature.

Model	Sensitivity (95% CI) (%)	Specificity (95% CI) (%)	Accuracy^a^ (95% CI) (%)	Predictive value^a^ (%)	
				PPV^b^ (95% CI)	NPV^c^ (95% CI)
Bayesian inference network	81.6 (65.7-92.3)	58.8 (32.9-81.6)	61.4 (48.5-74.2)	19.9 (10.5-29.2)	96.2 (92.5-100.0)
Information-theoretic set cover	0.0 (0.0-9.3)	100.0 (80.5-100.0)	88.9 (80.6-97.2)	0.0	88.9
Distance metric learning	84.2 (68.7-94.0)	64.7 (38.3-85.8)	66.9 (54.4-79.3)	23.0 (11.3-34.7)	97.0 (93.8-100.0)
Multinomial logistic regression ensemble	81.6 (65.7-92.3)	70.6 (44.0-89.7)	71.8 (59.9-83.7)	25.8 (11.4-40.2)	96.8 (93.7-100.0)
Logistic regression (Menni et al [14])	15.8 (6.0-31.3)	100.0 (80.5-100.0)	90.6 (82.9-98.3)	100.0	90.5 (88.7-92.2)

^a^Prevalence-adjusted metrics assume a COVID-19 prevalence of 11.1%.

^b^PPV: positive predictive value.

^c^NPV: negative predictive value.

### Incorporation of Location and Diagnostic Test Sequences

**Figure 1:**
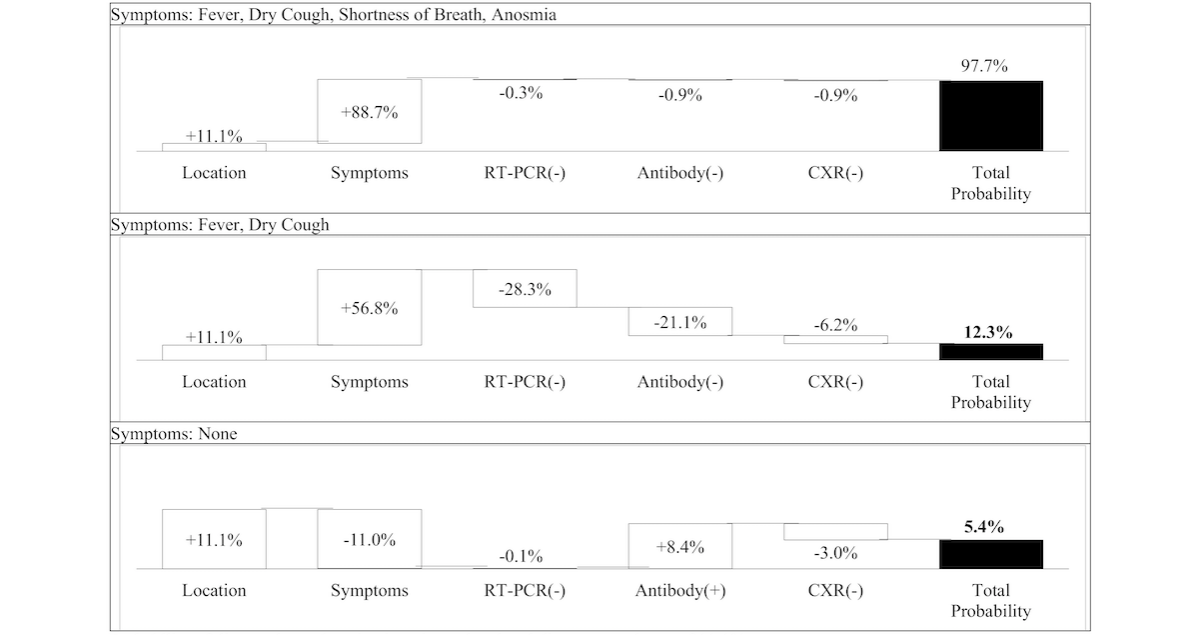
Probability of SARS-CoV-2 infection for common patient presentations and clinical test sequences. Probability of disease due to location is the estimated national disease prevalence of 11.1%. RT-PCR: reverse transcription-polymerase chain reaction; CXR: chest radiography.

We then employed the BN model to evaluate 3 hypothetical patients with 3 different presentations: (1) fever, dry cough, shortness of breath, and anosmia; (2) fever and dry cough; and (3) asymptomatic. We assumed all of these patients presented for care in an area with a local disease prevalence equivalent to the national disease prevalence of 11.1%. For patient 1, we simulated a clinically plausible imaging and test result sequence of negative RT-PCR, negative antibody, and negative CXR. The probability of a COVID-19 diagnosis following symptom collection was 99.8%. Despite negative test results, residual risk due to local disease prevalence and symptoms remained at 97.7%. The change in diagnosis probability, or the reduction in diagnostic uncertainty, was only 2.1% following all 3 negative tests. For patient 2, we simulated the same negative test sequence. In this scenario, the combination of negative test results with nonspecific symptom information resulted in a decrease in residual risk to 12.3%. The reduction in diagnostic uncertainty due to test results was 55.6%, primarily due to negative RT-PCR and negative antibody test results. The negative CXR provided less information as the reduction in diagnostic uncertainty following negative RT-PCR and antibody tests was only 6.2%. For patient 3, we simulated an imaging and test result sequence of negative RT-PCR, positive antibody, and negative CXR. The negative RT-PCR test reduced disease probability by only 0.1%, and the positive antibody test increased the probability of a COVID-19 diagnosis by 8.4%. The CXR results reduced diagnostic uncertainty by 3.0% (Figure 1).

**Figure 2:**
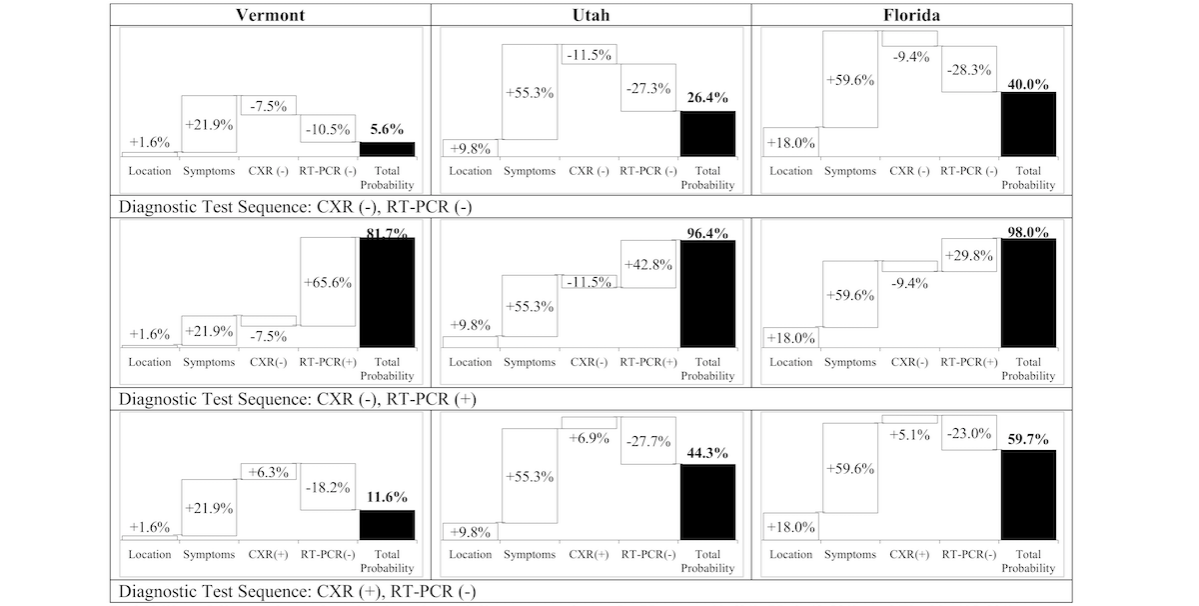
Impact of patient location and diagnostic test results on probability of SARS-CoV-2 infection. Prior probability of disease due to location is the estimated disease prevalence for Vermont (1.6%), Utah (9.8%), and Florida (18.0%). Incremental probability due to symptoms assumes the patient presents with only fever and dry cough. RT-PCR: reverse transcription-polymerase chain reaction; CXR: chest radiography.

To illustrate the dependence of risk assessment on local disease prevalence, we simulated a patient with symptoms of only fever and dry cough presenting in 3 locations with significantly different COVID-19 prevalence estimates: Vermont with an estimated statewide prevalence of 1.6%, Utah with an estimated statewide prevalence of 9.8%, and Florida with an estimated statewide prevalence of 18.0% at the time of the simulation. We combined results from 3 common test sequences with our BN pretest probabilities to compute location-dependent risk trajectories. The test sequences included: (1) negative CXR and negative RT-PCR; (2) negative CXR and positive RT-PCR; and (3) positive CXR and negative RT-PCR. Our results indicate that for a pauci-symptomatic patient presenting with identical symptoms in states with significantly different disease prevalence, the posttest probabilities of SARS-CoV-2 infection following common diagnostic test sequences demonstrate marked variation. Moreover, changes in diagnostic probability or reductions in diagnostic uncertainty are highly context and test dependent (Figure 2).

## Discussion

### Principal Findings

Our results suggest simple computable models that quantify patient risk of SARS-CoV-2 infection based on key elements of the clinical case can reduce diagnostic uncertainty for providers attempting to rule in or rule out disease with limited or conflicting information.

Building on work by Chishti et al [16], we chose probabilistic models considering the scarcity of detailed, individual patient data and to take advantage of the depth of published literature on aggregate symptom probabilities. Clinicians are trained in evidence-based medicine, and Bayesian reasoning provides a natural framework to understand the impact of incremental information on diagnosis probabilities. Our approaches to making stepwise diagnostic assessments with incremental information mimic clinical workflows and reflect the need for transparency and accommodation of new information critical to clinical decision making. As in Menni et al [14], we chose clinical indicators that would be easily obtained by patients and providers as well as predictive models that are easily computed and transparent to all users. While other machine learning approaches, such as generative adversarial networks, transfer learning, n-shot learning, and prototypical networks, are also robust for limited data, these methods can be opaque and inaccessible to providers and may be inflexible and fragile in an evolving clinical context.

Our most simple model, the Bayesian inference network, is transparent, easily interpreted, and highly modifiable depending on the user’s prior beliefs about location-based prevalence, conditional symptom probabilities, and imaging and laboratory test accuracy. Clinicians, educated in evidence-based medicine and often familiar with Bayesian decision making in diagnostic testing, are ideal users of this model. By developing base models that do not require access to large amounts of patient-level data and can accommodate changes in local provider beliefs and new sources of information, we alert physicians to the utility of using Bayesian reasoning to not only combine multiple data streams in order to make more informed diagnostic decisions but also to guide decisions about use of imaging and testing that will most effectively reduce diagnostic uncertainty.

### Limitations

Our study has limitations. First, we used simulated patient data based on prevalence and conditional symptom probabilities to train and validate our DML and ensemble models that biased the ensemble model to heavily weight the DML model predictions. Second, the number of patients in our clinical test data set was relatively small, and this data set was enriched for SARS-CoV-2–positive patients due to the cancellation of all elective procedures and the use of telemedicine for almost all patient visits during the study period, leaving clinics and hospitals open primarily for COVID-19 patients and the acutely ill. Third, 80% of the patients in our clinical test data set were from inpatient services, potentially biasing model accuracy by disease severity. Fourth, we chose as a reference standard the RT-PCR test results for SARS-CoV-2 infection despite outstanding questions about false negative rates in NAAT tests due to operator dependency and patient-level differences in viral loads across upper respiratory tract sites [4,27].

### Conclusions

Overall, we found that the Bayesian inference network, the metric learning model, and ensemble models trained and validated on a simulated patient data set had sensitivities (81.6%-84.2%) and specificities (58.8%-70.6%) for discriminating between COVID-19 infection and other potential diagnoses in real clinical settings. These models had higher sensitivities than reported for most commonly used diagnostics, and model specificities were higher than those of both imaging modalities. For purposes of comparison, the logistic regression model proposed by Menni et al [14], when applied to our clinical test data set, had a sensitivity of 15.8% and a specificity of 100.0%. Finally, our BN model shows that information acquired by imaging and testing choices is highly dependent on location and symptoms, and emphasizes the utility of a quantitative framework to guide clinical decision making in rapidly changing local environments with potentially unreliable diagnostic tests.

## Appendix

Multimedia Appendix 1 Supplemental materials.

## Abbreviations

BN: Bayesian network
CDC: Centers for Disease Control and Prevention
CDSS: clinical decision support systems
CT: computed tomography
CXR: chest radiography
DML: distance metric learning
NAAT: nucleic acid amplification test
NPV: negative predictive value
PPV: positive predictive value
RT-PCR: reverse transcription-polymerase chain reaction
SC: set-cover model
