# Supplementary material for: Computing SARS-CoV-2 Infection Risk From Symptoms, Imaging, and Test Data: Diagnostic Model Development
Source: J Med Internet Res. 2020 Dec 16;22(12):e24478. doi: 10.2196/24478 (PMC7746395; doi:10.2196/24478)
Supplement: Multimedia Appendix 1 [file jmir_v22i12e24478_app1.docx]

**TABLE OF CONTENTS**

**Supplemental Methods 1: Model Selection**

**Table S1.** Diagnosis Prevalence (%) and Conditional Symptom Probabilities (%) for SARS-CoV-2 Infection and Alternate Diagnoses.

**Summary of Patient Characteristics**

**Table S2.** Clinical Test Dataset: Individual Patient Characteristics.

**Figure S1.** Base Model Development: Bayesian Inference Network (BN) and Information-theoretic Set Cover (SC) Model

**Figure S2.** Base Model Development: Distance Metric Learning (DML) Model

**Figure S3.** Distance Metric Learning (DML) Model: Principal Axis Weights for Learned LMNN Linear Transformation

**Figure S4.** Distance Metric Learning (DML) Model: KNN Cross-Validation Accuracies by Neighbor Parameter on Simulated Patient Data

**Figure S5.** Ensemble Model Development

**Figure S6.** Ensemble Model Development: Ensemble Model Development: Average Cross-Validation Accuracies Across Diagnostic Classes by Training Fold on Simulated Patient Data

**Figure S7.** Base and Ensemble Model Testing: Clinical Test Dataset

**Supplemental Methods 1: Model Selection**

We selected COVID-19 diagnostic algorithms based on their ability to perform without access to large databases of patient records, the need for transparency in model computations, and the requirement of a simple, flexible framework able to assimilate new data.^[[1]](#endnote-1),^^[[2]](#endnote-2)^ We employed Bayesian inference networks (BNs) as they require no individual patient records to train, are adaptable to local knowledge of disease and symptom probabilities, and are familiar to many clinicians.^[[3]](#endnote-3)^ Similarly, set-cover models (SCs) require no granular training data and have been used previously for expert-based diagnostic systems.^[[4]](#endnote-4)^,^[[5]](#endnote-5)^ Finally, distance metric-learning models (DMLs) have been applied to clinical reasoning by researchers^[[6]](#endnote-6)^ and companies developing artificial intelligence for healthcare,^[[7]](#endnote-7)^ and logistic regression models are widely-used tool in clinical research efforts that prioritize interpretability of the relationships between patient characteristics and outcomes.^[[8]](#endnote-8)^

**Bayesian Inference Network**

Our BN model consisted of a causal diagnostic node (identified by infectious agent) and thirteen dependent symptom nodes that were conditionally independent given the diagnosis. The probability distribution over states in the causal node was based on mutually exclusive diagnosis prevalence. The conditional probability tables of each symptom node given a causal diagnosis were based on literature review. Using exact inference, we computed the causal node probability distribution over all potential diagnostic states for each patient given the diagnosis prevalence and that patient’s symptoms. We designed the classifier for this model to assign a binary label: COVID-19 infection vs. Other, depending on the maximum inferred diagnosis probability. If the diagnosis with the maximum inferred disease probability was COVID-19, this was labeled as “COVID-19 infection.” If COVID-19 did not have the maximum inferred disease probability, this was labeled as “Other” (Figure S1, Figure S7).

**Information-theoretic Set Cover Model**

We used disease prevalence and conditional symptom probabilities to compute the information gain of each symptom relative to the information encoded by the disease prevalence data. For each potential diagnosis, we weighted each symptom by its corresponding information gain and conditional probability and calculated the sum of all symptom weights for each potential diagnosis. The potential diagnosis with the highest weight for each patient was assigned as the most likely diagnosis, and the potential diagnosis with the lowest weight was the least likely diagnosis. We then projected these diagnosis weights into probability space by assigning the minimum weight diagnosis a probability of 0.0% and normalizing the differences between all diagnosis weights and the minimum diagnosis weight by the total sum of these weight differences. We transformed these diagnosis probabilities into classification labels using the same criteria as in our BN model (Figure S1, Figure S7).

**Distance Metric-Learning Model**

Without access to large patient record datasets, simulated symptom profiles and diagnoses for 100,000 patients using literature-review and current epidemiological data for prevalence and conditional symptom probabilities. We randomly assigned positive symptoms to patients within each disease class at a frequency identical to the conditional symptom probabilities for that disease. Using this synthesized dataset, we learned a largest margin nearest neighbor distance metric (Figure S2, Figure S3).^^[[9]](#endnote-9)^^ We applied this learned linear transformation to our synthesized dataset and used stratified, 5-fold cross validation on this synthesized dataset, to optimize our weighted, Euclidean-distance k-nearest-neighbor (KNN) classifier over k = [1, 3, 5, 7, 9, 11, 13, 15] (Figure S4). Our DML model includes both the learned linear transformation and the optimized KNN classifier. We then transformed our clinical test dataset using the learned linear transformation and applied the optimized KNN to obtain predicted diagnosis probabilities. We converted our diagnosis probabilities into classification labels using the same criteria as used in our BN model (Figure S7).

**Development of the Ensemble Model**

We applied our three base models (BN, SC, DML) to our synthesized dataset, and we used the predicted diagnosis probabilities of all of these models to train our multinomial logistic regression ensemble model using stratified, 5-fold cross validation (Figure S5). We applied the learned multinomial logistic regression coefficients in our ensemble model to the clinical test dataset (Figure S6) and computed the probability of each disease for every patient. We converted these probabilities into classification labels using the same criteria as in the BN model (Figure S7).

**Incorporating Location and Diagnostic Test Sequences**

We incorporated local disease prevalence into our BN model to create location-dependent pre-test probabilities from location and symptom information. We combined these location-specific pre-test probabilities with diagnostic test likelihood ratios to compute post-test risk trajectories following clinical imaging and test sequences for patients in common clinical scenarios. We modeled the impact of multiple diagnostic tests in sequence by multiplying likelihood ratios and computing the changes in diagnostic probability following each test result.

We chose to model symptom profiles of patients likely to be encountered by providers and imaging and test sequences commonly available to providers when diagnosing these patients. We simulated three different patient symptom profiles and examined the changes in diagnosis probability at each step of an imaging and test sequence to measure the symptom-dependent reduction in diagnostic uncertainty for each unique patient profile and test result. Next, we computed the differential diagnosis probabilities for a single patient as if she or he had presented to providers in three different U.S. states: Vermont (VT), Utah (UT) and Florida (FL). We calculated the location-dependent changes in diagnosis probability for three different sequences of imaging and test results in all locations (Figure 2).

**Table S1.** Diagnosis Prevalence (%) and Conditional Symptom Probabilities (%) for SARS-CoV-2 Infection and Alternate Diagnoses.

|  |  | **Diagnoses (Infectious Agents)** | | | | | | | | | | | |
| --- | --- | --- | --- | --- | --- | --- | --- | --- | --- | --- | --- | --- | --- |
|  |  | **SARS-CoV-2** | **hMPV** | **IV** | **PIV** | **RSV** | **RV** | **LD** | **MP** | **SP.** | **ADV** | **IM** | **Other** |
|  | Prevalence | 11.1^^[[10]](#endnote-10)^,^^^[[11]](#endnote-11)^,^^[[12]](#endnote-12)^^,^[[13]](#endnote-13)^,^^^[[14]](#endnote-14)^^ | 6.4^[[15]](#endnote-15)^ | 14.4^[[16]](#endnote-16)^ | 4.0^[[17]](#endnote-17)^ | 7.0^[[18]](#endnote-18)^ | 4.9^[[19]](#endnote-19)^ | 0.001^[[20]](#endnote-20)^ | 0.6^[[21]](#endnote-21),^^[[22]](#endnote-22)^ | 0.007^[[23]](#endnote-23)^ | 22.2^[[24]](#endnote-24)^ | 2.7^[[25]](#endnote-25)^ | 32.4 |
| **Symptoms** | Fever | 77.1^[[26]](#endnote-26)^ | 39.015 | 89.00^[[27]](#endnote-27)^ | 22.017 | 68.6^[[28]](#endnote-28)^ | 50.548 | 90.1^[[29]](#endnote-29)^ | 94.0^[[30]](#endnote-30)^ | 82.4^[[31]](#endnote-31)^ | 58.848 | 27.0^[[32]](#endnote-32)^ | 1.0 |
|  | Dyspnea | 56.526 | 46.315 | 58.0^[[33]](#endnote-33)^ | 42.0^[[34]](#endnote-34)^ | 35.0^[[35]](#endnote-35)^ | 1.0 | 45.429 | 1.0 | 75.231 | 1.0 | 1.0 | 1.0 |
|  | Dry cough | 62.0^[[36]](#endnote-36),^^[[37]](#endnote-37),^^[[38]](#endnote-38)^ | 97.615 | 97.027 | 67.017 | 76.035 | 80.648 | 81.229 | 99.030 | 86.631 | 88.248 | 40.032 | 1.0 |
|  | Productive cough | 29.836^,^37^,^38 | 53.715 | 63.2^[[39]](#endnote-39)^ | 44.334 | 72.828 | 74.0^[[40]](#endnote-40)^ | 47.229 | 45.030 | 52.0^[[41]](#endnote-41)^ | 73.040 | 1.0 | 1.0 |
|  | Fatigue | 39.136^,^37^,^38 | 75.615 | 89.027 | 3.434 | 7.628 | 10.0^[[42]](#endnote-42)^ | 75.029 | 57.5^[[43]](#endnote-43)^ | 92.331 | 83.040 | 93.032 | 1.0 |
|  | Loss of smell | 65.0^[[44]](#endnote-44)^ | 2.3^[[45]](#endnote-45)^* | 5.245* | 1.545* | 2.545* | 25.0^[[46]](#endnote-46)^ | 1.0 | 15.043 | 1.0 | 8.145* | 12.545* | 1.0 |
|  | Sore throat | 9.436^,^37^,^38 | 51.215 | 80.027 | 30.017 | 94.035 | 42.042 | 1.0 | 54.030 | 1.0 | 76.548 | 82.5^[[47]](#endnote-47)^ | 1.0 |
|  | Body / Muscle aches | 27.226 | 70.8^[[48]](#endnote-48)^ | 75.027 | 1.734 | 21.628 | 59.248 | 26.929 | 15.043 | 14.0^[[49]](#endnote-49)^ | 59.248 | 21.047 | 1.0 |
|  | Headache | 8.636^,^37^,^38 | 75.048 | 69.027 | 4.034 | 29.228 | 75.748 | 31.929 | 66.030 | 13.049 | 75.748 | 60.032 | 1.0 |
|  | Diarrhea | 23.726 | 1.0 | 17.027 | 9.234 | 2.228 | 17.040 | 26.629 | 15.030 | 11.049 | 33.040 | 1.0 | 1.0 |
|  | Nausea | 10.236 | 1.0 | 41.027 | 1.0 | 1.528 | 1.0 | 25.829 | 29.030 | 1.0 | 46.0^[[50]](#endnote-50)^ | 60.047 | 1.0 |

|  | Vomiting | 10.236 | 1.0 | 25.027 | 8.034 | 2.528 | 1.0 | 25.829 | 29.030 | 1.0 | 46.050 | 1.0 | 1.0 |
| --- | --- | --- | --- | --- | --- | --- | --- | --- | --- | --- | --- | --- | --- |
|  | Nasal congestion / Rhinorrhea | 8.437^,^38 | 85.415 | 76.027 | 70.017 | 88.035 | 17.042 | 1.0 | 29.030 | 1.0 | 9.1^[[51]](#endnote-51)^ | 47.032 | 1.0 |

Alternate diagnoses: human metapneumovirus (hMPV), influenza (IV), parainfluenza (PIV), respiratory syncytial virus (RSV), Legionnaires' disease (LD), mycoplasma pneumonia (MP), streptococcal pneumonia (SP), adenovirus (ADV), infectious mononucleosis (IM). * Prevalence-adjusted estimate of post-viral olfactory disorders following upper respiratory tract infection.

**Summary of Patient Characteristics**

Of 38 positive cases, 86.8% presented with fever, 86.8% with shortness of breath or dyspnea, 73.7% with non-productive cough, 10.5% with productive cough, 55.3% with fatigue or exhaustion, 13.2% with loss of smell, 2.6% with sore throat or pharyngalgia, 21.1% with body or muscle aches, 15.8% with headaches, 13.2% with diarrhea, 7.9% with nausea and 2.6% with vomiting.

Of the 17 negative cases, 58.8% presented with fever, 11.8% with shortness of breath or dyspnea, 11.8% with dry cough, 47.1% with productive cough, 41.2% with fatigue or exhaustion, 17.6% with sore throat or pharyngalgia, 11.8% with body or muscle aches, 17.6% with headache, 23.5% with diarrhea, 29.4% with nausea, 11.8% with vomiting, and 11.8% with nasal congestion or rhinorrhea”

**Table S2.** Clinical Test Dataset: Individual Patient Characteristics.

**Symptoms:**

1: Fever

2: Shortness of breath / Dyspnea

3: Dry cough

4: Productive cough

5: Fatigue or exhaustion

6: Loss of smell

7: Sore throat / Pharyngalgia

8: Body / Muscle Aches

9: Headache

10: Diarrhea

11: Nausea

12: Vomiting

13: Nasal congestion / Rhinorrhea

|  |  |  |  | **Symptoms** | | | | | | | | | | | | |
| --- | --- | --- | --- | --- | --- | --- | --- | --- | --- | --- | --- | --- | --- | --- | --- | --- |
| **Care Setting** | **Age** | **Sex** | **SARS-CoV-2** | 1 | 2 | 3 | 4 | 5 | 6 | 7 | 8 | 9 | 10 | 11 | 12 | 13 |
| Inpatient | 70-80 | F | (+) | Y | Y | Y | N | N | N | N | N | N | N | N | N | N |
| Inpatient | >80 | F | (+) | Y | Y | N | Y | Y | N | N | N | N | N | N | N | N |
| Inpatient | 70-80 | F | (+) | Y | Y | N | N | Y | N | N | N | N | N | N | N | N |
| Inpatient | 60-70 | M | (+) | N | Y | Y | Y | N | N | N | N | N | Y | N | N | N |
| Inpatient | >80 | M | (+) | Y | Y | Y | N | N | N | N | N | N | N | N | N | N |
| Inpatient | 70-80 | M | (+) | Y | Y | Y | N | N | N | N | N | N | N | N | N | N |
| Inpatient | 60-70 | M | (+) | Y | N | Y | N | N | N | N | Y | N | N | N | N | N |
| Inpatient | <60 | F | (+) | Y | Y | Y | N | N | N | N | N | N | Y | N | N | N |
| Inpatient | <60 | M | (+) | Y | N | Y | N | Y | N | N | Y | Y | Y | Y | Y | N |
| Inpatient | >80 | F | (+) | Y | Y | Y | N | N | N | N | N | N | N | N | N | N |
| Inpatient | 60-70 | M | (+) | Y | N | N | N | N | N | N | N | N | N | N | N | N |
| Inpatient | 60-70 | M | (-) | Y | N | Y | Y | Y | N | N | N | N | Y | Y | N | N |
| Inpatient | <60 | F | (-) | Y | N | N | N | N | N | Y | N | N | N | N | N | N |
| Inpatient | 60-70 | F | (-) | N | N | N | N | N | N | N | N | Y | N | Y | N | N |
|  |  |  |  | **Symptoms** | | | | | | | | | | | | |
| **Care Setting** | **Age** | **Sex** | **SARS-CoV-2** | 1 | 2 | 3 | 4 | 5 | 6 | 7 | 8 | 9 | 10 | 11 | 12 | 13 |
| Inpatient | <60 | M | (-) | Y | Y | N | N | Y | N | N | N | N | N | N | N | N |
| Inpatient | 70-80 | F | (-) | Y | Y | N | Y | Y | N | N | N | N | N | N | N | Y |
| Inpatient | <60 | F | (-) | Y | N | N | Y | Y | N | N | N | N | N | N | N | N |
| Outpatient | <60 | M | (-) | Y | N | N | N | Y | N | Y | N | Y | N | Y | N | N |
| Outpatient | <60 | F | (-) | N | N | Y | N | N | N | N | N | N | N | N | N | N |
| Outpatient | 70-80 | F | (-) | N | N | N | Y | N | N | N | N | N | N | N | N | N |
| Outpatient | <60 | F | (-) | N | N | N | Y | N | N | N | N | N | N | N | N | N |
| Outpatient | <60 | F | (-) | Y | N | N | Y | Y | N | N | Y | N | N | N | N | N |
| Outpatient | <60 | M | (+) | Y | Y | Y | N | Y | N | N | Y | Y | N | N | N | N |
| Outpatient | <60 | F | (+) | Y | Y | Y | N | Y | N | N | N | N | N | N | N | N |
| Outpatient | <60 | M | (+) | Y | Y | Y | N | Y | N | Y | Y | Y | N | N | N | N |
| Outpatient | <60 | F | (+) | N | N | N | N | N | N | N | N | Y | N | N | N | N |
| Outpatient | <60 | M | (+) | N | Y | N | N | Y | N | N | N | N | N | N | N | N |
| Outpatient | <60 | F | (+) | N | Y | Y | N | Y | N | N | Y | N | N | N | N | N |
|  |  |  |  | **Symptoms** | | | | | | | | | | | | |
| **Care Setting** | **Age** | **Sex** | **SARS-CoV-2** | 1 | 2 | 3 | 4 | 5 | 6 | 7 | 8 | 9 | 10 | 11 | 12 | 13 |
| Inpatient | <60 | M | (+) | Y | Y | Y | N | Y | N | N | Y | N | N | N | N | N |
| Inpatient | 60-70 | M | (-) | N | N | N | N | N | N | N | N | N | Y | Y | Y | N |
| Inpatient | <60 | M | (+) | Y | Y | N | Y | Y | N | N | N | N | N | Y | N | N |
| Inpatient | 70-80 | M | (+) | Y | Y | Y | N | N | N | N | N | N | N | N | N | N |
| Inpatient | <60 | F | (+) | Y | Y | N | N | Y | N | N | N | N | N | N | N | N |
| Inpatient | <60 | F | (+) | Y | Y | Y | N | N | N | N | N | N | N | N | N | N |
| Inpatient | <60 | M | (+) | Y | Y | Y | N | N | N | N | N | N | N | N | N | N |
| Inpatient | 60-70 | F | (+) | Y | Y | N | N | Y | Y | N | N | N | N | N | N | N |
| Inpatient | <60 | M | (+) | Y | Y | Y | N | Y | N | N | N | N | N | N | N | N |
| Inpatient | 60-70 | F | (+) | N | Y | Y | N | Y | N | N | N | N | N | N | N | N |
| Inpatient | <60 | F | (+) | Y | Y | Y | N | N | N | N | N | N | N | N | N | N |
| Inpatient | 60-70 | M | (+) | Y | Y | Y | N | Y | Y | N | N | N | Y | N | N | N |
| Inpatient | 60-70 | M | (+) | Y | N | N | N | Y | Y | N | N | N | N | N | N | N |
| Inpatient | <60 | F | (+) | Y | Y | Y | N | Y | Y | N | N | N | N | N | N | N |
|  |  |  |  | **Symptoms** | | | | | | | | | | | | |
| **Care Setting** | **Age** | **Sex** | **SARS-CoV-2** | 1 | 2 | 3 | 4 | 5 | 6 | 7 | 8 | 9 | 10 | 11 | 12 | 13 |
| Inpatient | <60 | M | (+) | Y | Y | Y | N | Y | N | N | Y | N | N | N | N | N |
| Inpatient | >80 | F | (+) | Y | Y | Y | Y | Y | N | N | N | N | N | N | N | N |
| Inpatient | 60-70 | M | (+) | Y | Y | Y | N | N | N | N | N | Y | Y | N | N | N |
| Inpatient | 60-70 | M | (+) | Y | Y | N | N | N | N | N | N | Y | N | N | N | N |
| Inpatient | >80 | F | (+) | Y | Y | Y | N | N | N | N | N | N | N | N | N | N |
| Inpatient | 70-80 | F | (+) | Y | Y | Y | N | N | Y | N | N | N | N | N | N | N |
| Inpatient | <60 | M | (+) | Y | Y | Y | N | Y | N | N | N | N | N | N | N | N |
| Inpatient | <60 | M | (+) | Y | Y | Y | N | Y | N | N | Y | N | N | Y | N | N |
| Inpatient | >80 | F | (-) | Y | N | N | N | N | N | N | N | N | N | N | N | N |
| Inpatient | <60 | M | (-) | Y | N | N | Y | N | N | N | N | N | N | N | N | N |
| Inpatient | 70-80 | F | (-) | N | N | N | N | Y | N | Y | Y | Y | Y | Y | Y | N |
| Inpatient | 60-70 | F | (-) | N | N | N | Y | N | N | N | N | N | N | N | N | Y |
| Inpatient | 70-80 | M | (-) | Y | N | N | N | N | N | N | N | N | Y | N | N | N |

**Figure S1.** Base Model Development: Bayesian Inference Network (BN) and Information-theoretic Set Cover (SC) Model


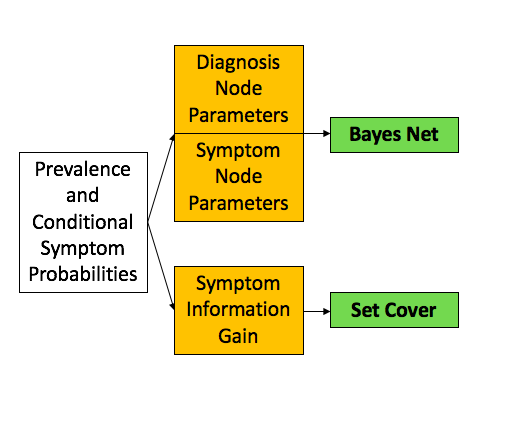


**Figure S2.** Base Model Development: Distance Metric Learning (DML) Model


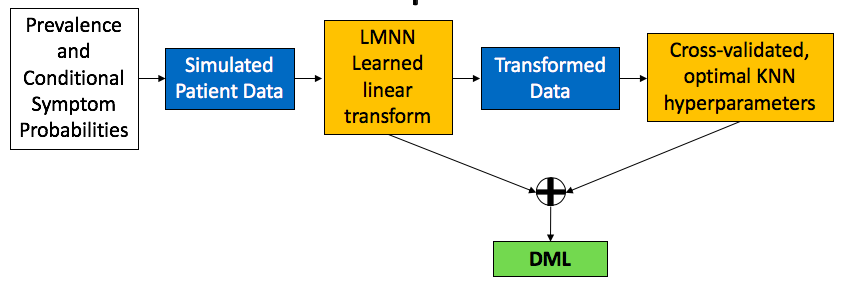


l

**Figure S3.** Distance Metric Learning (DML) Model: Principal Axis Weights for Learned LMNN Linear Transformation

**Figure S4.** Distance Metric Learning (DML) Model: KNN Cross-Validation Accuracies by Neighbor Parameter on Simulated Patient Data

**Figure S5.** Ensemble Model Development


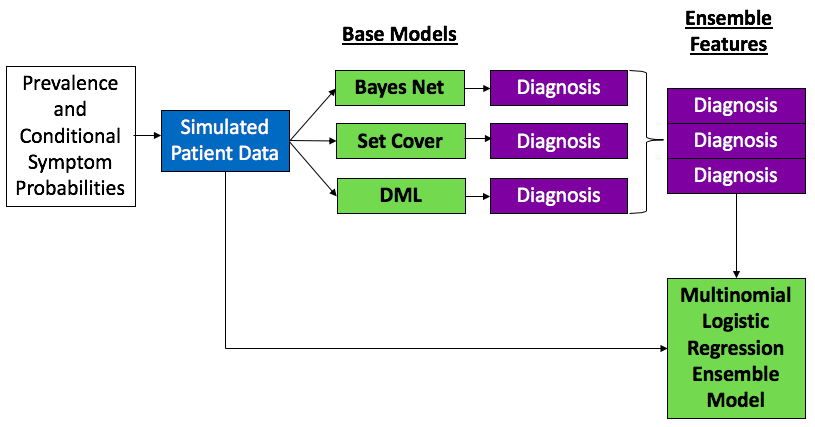


**Figure S6.** Ensemble Model Development: Average Cross-Validation Accuracies Across Diagnostic Classes by Training Fold on Simulated Patient Data

**Figure S7.** Base and Ensemble Model Testing: Clinical Test Dataset

**
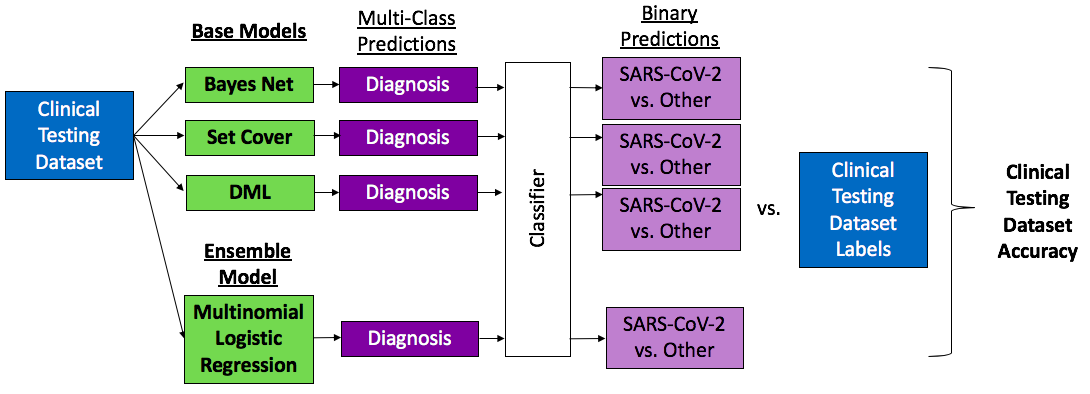
**

1. . Ahmad MA, Eckert C, Teredesai A. Interpretable Machine Learning in Healthcare. In: Proceedings of the 2018 ACM International Conference on Bioinformatics, Computational Biology, and Health Informatics. New York, NY, USA: ACM 2018; p. 559–60. [↑](#endnote-ref-1)
2. . Bates DW, Kuperman GJ, Wang S, et al. Ten Commandments for Effective Clinical Decision Support: Making the Practice of Evidence-based Medicine a Reality. JAMIA 2003; 10(6):523–30. [↑](#endnote-ref-2)
3. . Andreassen S, Jensen F V, Olesen KG. Medical expert systems based on causal probabilistic networks. Int J Biomed Comput 1991;28(1):1–30. [↑](#endnote-ref-3)
4. . Reggia JA, Nau DS, Wang PY. Diagnostic expert systems based on a set covering model. Intl J Man-Machine Stud 1983;19(5):437-60. [↑](#endnote-ref-4)
5. . Müller L, Gangadharaiah R, Klein SC, et al. An open access medical knowledge base for community driven diagnostic decision support system development. BMC Med Inform Decis Ma. 2019 Apr 27;19(1):93. [↑](#endnote-ref-5)
6. . Gottlieb A, Stein GY, Ruppin E, Altman RB, Sharan R. A method for inferring medical diagnoses from patient similarities. BMC Med 2013;11(1):1-0. [↑](#endnote-ref-6)
7. . Wang F, Hu J, Sun J. Medical prognosis based on patient similarity and expert feedback. InProceedings of the 21st International Conference on Pattern Recognition (ICPR2012) 2012 Nov 11 (pp. 1799-1802). IEEE. [↑](#endnote-ref-7)
8. . Tu JV. Advantages and disadvantages of using artificial neural networks versus logistic regression for predicting medical outcomes. J Clin Epi 1996;49(11):1225-31. [↑](#endnote-ref-8)
9. . Weinberger KQ, Saul LK. Distance metric learning for large margin nearest neighbor classification. Journal of Machine Learning Research 2009;10(2). [↑](#endnote-ref-9)
10. . Dong E, Du H, Gardner L. An interactive web-based dashboard to track COVID-19 in real time. Lancet Infect. Dis 2020;20(5):533–4. [↑](#endnote-ref-10)
11. . State Population Totals and Components of Change: 2010-2019. Washington, DC: United States Census Bureau, December 2019. (https://www.census.gov/data/tables/time-series/demo/popest/2010s-state-total.html) [↑](#endnote-ref-11)
12. . Commercial Laboratory Seroprevalence Data. Atlanta, GA: CDC, June 2020. (https://www.cdc.gov/coronavirus/2019-ncov/cases-updates/commercial-lab-surveys.html) [↑](#endnote-ref-12)
13. . Havers FP, Reed C, Lim TW, et al. Seroprevalence of Antibodies to SARS-CoV-2 in Six Sites in the United States, March 23-May 3, 2020. medRxiv 2020;2020.06.25.20140384. [↑](#endnote-ref-13)
14. . Pei S, Shaman J. Initial Simulation of SARS-CoV2 Spread and Intervention Effects in the Continental US. medRxiv 2020;2020.03.21.20040303. [↑](#endnote-ref-14)
15. . Walsh EE, Peterson DR, Falsey AR. Human metapneumovirus infections in adults: Another piece of the puzzle. Arch Intern Med 2008;168(22):2489–96. [↑](#endnote-ref-15)
16. . 2019-2020 U.S. Flu Season: Preliminary Burden Estimates. Atlanta, GA: CDC, April 2020. (https://www.cdc.gov/flu/about/burden/preliminary-in-season-estimates.htm) [↑](#endnote-ref-16)
17. . Maykowski P, Smithgall M, Zachariah P, Oberhardt M, et al. Seasonality and clinical impact of human parainfluenza viruses. Influenza Other Respir Viruses 2018;12(6):706-716. [↑](#endnote-ref-17)
18. . Gonik B. The Burden of Respiratory Syncytial Virus Infection in Adults and Reproductive-Aged Women. Glob Heal Sci Pract 2019;7(4):515-520. [↑](#endnote-ref-18)
19. . Winther B, Gwaltney JM Jr, Mygind N, Hendley JO. Viral-induced rhinitis. Am J Rhinol. 1998;12(1):17-20. [↑](#endnote-ref-19)
20. . Active Bacterial Core Surveillance for Legionellosis - United States, 2011–2013. Atlanta, GA: CDC, October 2015. (https://www.cdc.gov/mmwr/preview/mmwrhtml/mm6442a2.htm) [↑](#endnote-ref-20)
21. . Marston BJ, Plouffe JF, File Jr TM, et al. Incidence of community-acquired pneumonia requiring hospitalization. Results of a population-based active surveillance study in Ohio. The Community-Based Pneumonia Incidence Study Group. Arch Intern Med 1997;157:1709–18. [↑](#endnote-ref-21)
22. . Porath A, Schlaeffer F, Lieberman D. The epidemiology of community-acquired pneumonia among hospitalized adults. J Infect 1997;34:41–8. [↑](#endnote-ref-22)
23. . Pneumococcal Disease: Surveillance and Reporting. Atlanta, GA: CDC, September 2017. (https://www.cdc.gov/pneumococcal/surveillance.html) [↑](#endnote-ref-23)
24. . Russell KL, Broderick MP, Franklin SE, et al. Transmission dynamics and prospective environmental sampling of adenovirus in a military recruit setting. J Infect Dis 2006;194(7):877-85. [↑](#endnote-ref-24)
25. . Womack J, Jimenez M. Common questions about infectious mononucleosis. Am Fam Physician 2015;91(6):372-376. [↑](#endnote-ref-25)
26. . Goyal P, Choi JJ, Pinheiro LC, et al. Clinical Characteristics of Covid-19 in New York City. N Engl J Med 2020;382(24):2372–4. [↑](#endnote-ref-26)
27. . Yang JH, Huang PY, Shie S Sen, et al. Predictive Symptoms and Signs of Laboratory-confirmed Influenza. Med (United States) 2015;94(44):e1952. [↑](#endnote-ref-27)
28. . Park WJ, Yoo SJ, Lee SH, Chung JW, Jang KH, Moon JD. Respiratory syncytial virus outbreak in the basic military training camp of the republic of Korea Air Force. J Prev Med Public Health 2015;48(1):10-7. [↑](#endnote-ref-28)
29. . Woodhead MA, Macfarlane JT. Legionnaires' disease: a review of 79 community acquired cases in Nottingham. Thorax 1986;41(8):635-640. [↑](#endnote-ref-29)
30. . Foy HM, Kenny GE, McMahan R, Mansy AM, Grayston JT. Mycoplasma pneumoniae pneumonia in an urban area. Five years of surveillance. JAMA 1970;214(9):1666-1672. [↑](#endnote-ref-30)
31. . Brandenburg JA, Marrie TJ, Coley CM, et al. Clinical presentation, processes and outcomes of care for patients with pneumococcal pneumonia. J Gen Intern Med 2000;15(9):638-646. [↑](#endnote-ref-31)
32. . Aronson MD, Komaroff AL, Pass TM, Ervin CT, Branch WT. Heterophil antibody in adults with sore throat: frequency and clinical presentation. Ann Intern Med 1982;96(4):505-508. [↑](#endnote-ref-32)
33. . Miller MR, Peters TR, Suerken CK, et al. Predictors of Influenza Diagnosis Among Patients With Laboratory-Confirmed Influenza. J Infect Dis. 2015;212(10):1604-1612. [↑](#endnote-ref-33)
34. . Liu WK, Liu Q, Chen DH, et al. Epidemiology and clinical presentation of the four human parainfluenza virus types. BMC Infect Di. 2013;13:28. [↑](#endnote-ref-34)
35. . O'Shea MK, Ryan MA, Hawksworth AW, Alsip BJ, Gray GC. Symptomatic respiratory syncytial virus infection in previously healthy young adults living in a crowded military environment. Clin Infect Dis 2005;41(3):311-317. [↑](#endnote-ref-35)
36. . Zhu J, Ji P, Pang J, et al. Clinical characteristics of 3062 COVID‐19 patients: A meta‐analysis. J Med Virol 2020;jmv.25884. [↑](#endnote-ref-36)
37. . Heydari K, Rismantab S, Shamshirian A, et al. Clinical and Paraclinical Characteristics of COVID-19 patients: A systematic review and meta-analysis. medRxiv 2020; 2020.03.26.20044057. [↑](#endnote-ref-37)
38. . Ma C, Gu J, Hou P, et al. Incidence, clinical characteristics and prognostic factor of patients with COVID-19: a systematic review and meta-analysis. medRxiv 2020;2020.03.17.20037572. [↑](#endnote-ref-38)
39. . Choi SH, Chung JW, Kim T, Park KH, Lee MS, Kwak YG. Late diagnosis of influenza in adult patients during a seasonal outbreak. Korean J Intern Med 2018;33(2):391–6. [↑](#endnote-ref-39)
40. . Jennings LC, Anderson TP, Beynon KA, et al. Incidence and characteristics of viral community-acquired pneumonia in adults. Thorax 2008;63(1):42-48. [↑](#endnote-ref-40)
41. . Lippmann ML, Goldberg SK, Walkenstein MD, Herring W, Gordon M. Bacteremic pneumococcal pneumonia. A community hospital experience. Chest 1995;108(6):1608-1613. [↑](#endnote-ref-41)
42. . Arruda E, Pitkäranta A, Witek TJ Jr, Doyle CA, Hayden FG. Frequency and natural history of rhinovirus infections in adults during autumn. J Clin Microbiol 1997;35(11):2864-8. [↑](#endnote-ref-42)
43. . Feizi T, Maclean H, Sommerville RG, Selwyn JG. Studies on an epidemic of respiratory disease caused by Mycoplasma pneumoniae. Br Med J 1967;1(5538):457-460. [↑](#endnote-ref-43)
44. . Menni C, Valdes AM, Freidin MB, et al. Real-time tracking of self-reported symptoms to predict potential COVID-19. Nat Med 2020;1–4. [↑](#endnote-ref-44)
45. . Nordin S, Brämerson A. Complaints of olfactory disorders: epidemiology, assessment and clinical implications. Curr Opin Allergy Clin Immunol 2008;8(1):10–5. [↑](#endnote-ref-45)
46. . Suzuki M, Saito K, Min WP, Vladau C, Toida K, Itoh H, Murakami S. Identification of viruses in patients with postviral olfactory dysfunction. Laryngoscope 2007;117(2):272-7. [↑](#endnote-ref-46)
47. . Rea TD, Russo JE, Katon W, Ashley RL, Buchwald DS. Prospective study of the natural history of infectious mononucleosis caused by Epstein-Barr virus. J Am Board Fam Pract 2001;14(4):234-242. [↑](#endnote-ref-47)
48. . Bellei N, Carraro E, Perosa A, et al. Acute respiratory infection and influenza-like illness viral etiologies in Brazilian adults. J Med Virol 2008;80(10):1824–7. [↑](#endnote-ref-48)
49. . Granados A, Podzamczer D, Gudiol F, Manresa F. Pneumonia due to Legionella pneumophila and pneumococcal pneumonia: similarities and differences on presentation. Eur Respir J 1989;2(2):130-134. [↑](#endnote-ref-49)
50. . Hamkar R, Yahyapour Y, Noroozi M, Nourijelyani K, Jalilvand S, Adibi L, Vaziri S, Poor-Babaei A, Pakfetrat A, Savad-Koohi R. Prevalence of Rotavirus, Adenovirus, and Astrovirus Infections among Patients with Acute Gastroenteritis in, Northern Iran. Iran J Public Health 2010;39(2):45-51. [↑](#endnote-ref-50)
51. . Koren MA, Arnold JC, Fairchok MP, Lalani T, Danaher PJ, Schofield CM, Rajnik M, Hansen EA, Mor D, Chen WJ, Ridoré M, Burgess TH, Millar EV. Type-specific clinical characteristics of adenovirus-associated influenza-like illness at five US military medical centers, 2009-2014. Influenza Other Respir Viruses 2016;10(5):414-20. [↑](#endnote-ref-51)
